# Repeated neurofilament light chain measurements did not capture Riluzole therapeutic effect in amyotrophic lateral sclerosis patients

**DOI:** 10.1111/cns.13894

**Published:** 2022-06-25

**Authors:** Florence Esselin, Elisa De la Cruz, Christophe Hirtz, Laurent Tiers, Sébastien Alphandery, Léandra Baudesson, Guillaume Taieb, William Camu, Sylvain Lehmann

**Affiliations:** ^1^ Explorations neurologiques et centre SLA Univ Montpellier, CHU Gui de Chauliac, INM, INSERM Montpellier France; ^2^ LBPC‐PPC, Univ Montpellier CHU Montpellier, INSERM, INM Montpellier Montpellier France

**Keywords:** ALS, NfL, Riluzole, Simoa, treatment

## Abstract

**Background:**

Little is known about the influence of Riluzole on serum neurofilament light chain (sNfL) levels, a biomarker of prognosis in amyotrophic lateral sclerosis (ALS), and variations with time of sNfL concentrations are controversial.

**Methods:**

Sera from ALS patients (*n* = 141) and controls (*n* = 33) were collected at inclusion (sNfL1) and second visit (sNfL2, mean delay 10.4 ± 8.7 months). sNfL levels, determined by single‐molecule array, were compared between ALS and controls at both time points. sNfL concentration changes were compared between patients with Riluzole (w/Ril) at inclusion in the study and those who were treated by Riluzole following inclusion (w/o Ril). The factors influencing sNfL concentrations and changes were studied using linear regression and multivariate analysis.

**Results:**

sNfL levels were higher in ALS patients than in controls at the two time points (*p* < 0.00001). In ALS patients, sNfL concentrations were higher in females for both sNfL1 (*p* = 0.014) and sNfL2 (*p* < 0.001). In the whole ALS group, sNfL levels were higher at sNfL2 than at sNfL1 (*p* < 0.001). sNfL1 and sNfL2 concentrations were similar between the two ALS subgroups (w/ and w/o Ril). ALS functional rating scale‐revised rate of decline and gender were the two main factors significantly influencing both sNfL1 and sNfL2 levels (*p* < 0.01). However, only gender was shown to significantly influence sNfL changes with time (*p* = 0.003).

**Conclusions:**

In this study, sNfL levels increased with time in ALS patients and there was no difference between subjects already treated by Riluzole and those treated after sNfL1. Further studies with larger population samples and different sampling intervals are warranted to better determine the real potential of sNfL measurement as a tool to monitor treatment response in ALS.

## Graphical abstract

NfL levels increased with time in ALS patients. Patients already under Riluzole and those without treatment had similar NfL concentrations at inclusion. After 1 year of treatment, the increase in NfL levels was of the same magnitude in both groups.

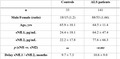

## 1 INTRODUCTION

ALS is a devastative neurodegenerative disorder involving upper and lower motoneurons (MNs), leading to death in a median time of 3 years after onset.^1^ Prognosis is highly variable, and diagnosis may be sometimes difficult to rapidly ascertain. Thus, there is a need for reliable and easily accessible biomarkers of the disease.^2^ Serum neurofilament light chain (sNfL) seems to comply with the requirement for a reliable biomarker for ALS as it is closely correlated with prognosis, even at first diagnostic referral, and accurately help distinguishing between ALS and its main differential diagnoses.^3^, ^4^, ^5^ sNfL is also an easily accessible measure as recent techniques, and particularly Simoa, may identify femtomolar levels of proteins, and serum and CSF concentrations have been shown to be highly correlated.^6^ An additional criterion for a reliable biomarker would be to accurately reflect the therapeutic effect of a drug. In ALS, the use of Tofersen that limits patients' worsening showed that sNfL levels decreased rapidly following the treatment setup and similar findings were made with Nusinersen, a treatment for spinal muscular atrophies.^7^, ^8^ To accurately interpret a treatment response, it is important to demonstrate that sNfL concentrations do not spontaneously vary with time, and data regarding the variation of serum and CSF NfL concentrations in ALS are scarce and not concordant.^9^, ^10^, ^11^, ^12^ Riluzole is the only worldwide marketed drug in ALS. This drug has proven effective in ALS, improving survival by 34% after 18 months of treatment in two consecutive phase II and III trials.^13^, ^14^ To date, no systematic study explored the effect of Riluzole on sNfL concentrations. We thus underwent a study to refine our knowledge on sNfL levels during the course of ALS and after Riluzole treatment.

## 2 METHODS

### 2.1 Patients and clinical characterization

All subjects were recruited in our tertiary ALS center where they were all referred to for suspicion of ALS. Demographics and clinical data were collected, including age at blood collection and gender. All ALS patients fulfilled the criteria of either probable or definite ALS according to international ALS criteria of El Escorial and Airlie House.^15^ Patients with diagnostic uncertainty, and particularly those with still suspected or possible ALS at the time of analysis, were excluded. For ALS patients, demographics and clinical data were collected at recruitment and during follow‐up and included age at onset of first weakness (ALS onset), age at blood collection, site of onset (bulbar, upper limb, or lower limb), usual body weight, and weight at each visit, ALS Functional Rating Scalerevised (ALSFRS‐r) scores.^16^ The last two parameters allowed to calculate a monthly rate of change for weight and ALSFRS‐r score, two important prognostic factors. Two blood samples for sNFL levels determination were done. sNfL1 was the first one, done at inclusion in the study, and sNfL2 was the second one, a few months later. ALS patients were subdivided into two groups to study the influence of Riluzole treatment. One group of patients was named “with Riluzole at sNfL1” (w/Ril), if, at the time of inclusion, those patients were already treated by Riluzole. The other group of ALS patients only started Riluzole after inclusion, for example, the day after sNfL1, and was named “w/o Riluzole at sNfL1” (w/o/Ril). However, all the patients from both groups were still treated by Riluzole at the second time point, sNfL2. Controls were all recruited at the first referral visit, in the same conditions as ALS, with two sets of blood collections, one at inclusion and the second at the end of their diagnostic process. This work was undertaken with the understanding and written consent of each subject, conformed with World Medical Association Declaration of Helsinki. The study was approved by Institutional review board of CHU Montpellier, France, reference: IRB‐MTP_2021_04_202100783.

### 2.2 Sample collection and analysis

Blood samples were collected at two different visits in 5‐ml dry tubes (Venosafe VF‐109SP, Terumo) and centrifuged, and aliquots were stored at −80°C until use.^17^ sNfL concentration was determined using commercial NfL assay kit (Quanterix, USA) based on ultrasensitive Simoa technology.^18^ All samples were fourfold diluted with the provided dilution buffer to minimize matrix effects. After dilution, the lowest limit of detection was 0.038 pg/ml and the limit of quantification was of 0.696 pg/ml. Quality controls with low sNfL concentration (QC 1 with mean concentration of 4.3 pg/ml) and QC high sNfL known concentration (QC 2 with mean concentration of 141.5 pg/ml) were provided in the kits. We observed a low inter‐assay variation for QC 1 and QC 2 with coefficient of variation (CV) of 5.8% and 3.5%, respectively. In addition to quality controls, one internal QC represented by pooled serum (mean sNfL of 13.0 pg/ml) was analyzed at the beginning and at the end in each sample plate with low intra‐assay and inter‐assay CV of 6% and 11%, respectively. This analysis confirmed the low inter‐assay variation for three serum samples with CV of 4.5%, 10% and 4.1% for sNfL. Detailed methodology and technique have been previously described.^3^

### 2.3 Statistical analysis

The patients' characteristics were reported and compared between ALS and controls but also in ALS patients' subgroups w/ and w/o/ Ril, and before and after Riluzole treatment. Quantitative variables were expressed by their mean ± standard deviation and the qualitative variables by their frequencies. After test for normality (Kolmogorov–Smirnov), non‐parametric tests, Mann–Whitney and Wilcoxon, were used for comparisons between groups for quantitative variables. Frequencies of qualitative variables between groups used chi‐square test. To study the impact of ALS parameters on sNfL levels at different time points, univariate linear regression was carried out. The variables with a *p*‐value lower than 0.10 were then considered for a multivariate model, and the variables with a *p*‐value lower than 0.05 in the multivariate model after a stepwise selection of variables were considered statistically significant and were reported in the table. The same procedure was applied for studying the influence of ALS parameters on sNfL changes. The type I error rate was 0.05. Statistical analysis was performed using XLSTAT statistical and data analysis solution (Addinsoft, Paris, France, https://www.xlstat.com).

## 3 RESULTS

### 3.1 In ALS sNfL levels increase with time and are significantly higher than controls

A total of 174 individuals were recruited, 106 men and 68 women. They all were analyzed for sNfL levels at two different time points; the first one corresponded to first referral (sNfL1) and the second one (sNfL2) to the second visit (mean delay 10.4 ± 8.7 months). Distribution of gender and mean age at first blood collection, between the ALS group (*n* = 141) and the control group (*n* = 33), were comparable (Table 1). The diagnoses and characteristics of controls are described in detail in Table S1. sNfL1 levels between controls and ALS patient were significantly different, 24.4 ± 18.1 pg/ml and 64.2 ± 47.2 pg/ml, respectively. The time interval between sNfL1 and sNfL2 was similar between the two groups, and sNfL2 concentrations remained significantly lower in controls (22.2 ± 17.8 pg/ml) compared with the ALS group (77.6 ± 66.3 pg/ml). sNfL1 and sNfL2 were not statistically different in controls. Conversely, sNfL2 was significantly increased compared with sNfL1 in the ALS group.

**TABLE 1:** Characteristics of ALS and controls

	Controls	ALS patients	*p*
*n*	33	141	
Male/Female (ratio)	18/15 (1.2)	88/53 (1.66)	ns
Age, years	65.9 ± 10.1	64.5 ± 11.4	ns
sNfL1, pg/ml	24.4 ± 18.1	64.2 ± 47.4	<0.0001
sNfL2, pg/ml	22.2 ± 17.8	77.6 ± 66.3	<0.0001
*p* (sNf1 vs. sNf2)	ns	<0.001	
Delay sNfL1 / sNfL2, months	9.7 ± 7.3	10.6 ± 9.0	ns
sNfL1 levels			
Men	23.4 ± 16.3	53.2 ± 28.4	<0.0001
Women	24.1 ± 19.2	82.4 ± 64.4	<0.0001
*p* (men vs. women)	ns	0.014	
sNfL2 levels			
Men	20.4 ± 12.1	60.0 ± 38.3	<0.0001
Women	22.6 ± 20.0	106.8 ± 89.4	<0.0001
*p* (men vs. women)	ns	<0.001	
Men, *p* (NfL1 vs. NfL2)	ns	ns	
Women, *p* (NfL1 vs. NfL2)	ns	ns	

*Note:* Values are mean ± SD, except for gender.

Abbreviations: ALS, amyotrophic lateral sclerosis; *n*, number of subjects; ns, not significant; sNfL—serum neurofilament light chain.

At both time points, sNfL1 and sNfL2, women with ALS had significantly higher sNfL concentrations than men, while there was no difference in controls regarding gender. Similarly, at both time points, sNfL levels were higher in ALS than controls, for men and for women. However, when comparing sNfL1 and sNfL2 according to gender in ALS patients, although concentrations at sNfL2 were higher in both groups, this did not reach statistical significance.

### 3.2 sNfL concentrations increase with time in both ALS subgroups w/ and w/o Riluzole

A total of 86 patients were already treated by Riluzole at inclusion in the study (w/Ril group), compared with 55 patients for whom Riluzole was started after inclusion (w/o Ril group). Between these two groups, gender, site of onset, age at onset, and usual weight were similar (Table 2 and Table S2). At entry, delay between ALS onset and sNfL1 measurement was longer in patients w/Ril (35.5 ± 35.6 months) compared with the other group (18.7 ± 43.6, *p* < 0.0001). ALSFRS‐R score was also lower in the w/Ril group: 36.8 ± 7.1 vs. 41.7 ± 5.2. However, age at inclusion, rate of ALSFRS‐R decline, rate of weight loss, and sNfL1 levels were similar. After the second measure of sNfL levels, two types of comparisons were done. First, we compared each group's (w/ and w/o Ril) ALS parameters between sNfL2 and sNfL1 time points. In both groups, ALSFRS‐R score worsened significantly as expected, and we also noted an increase in sNfL levels, by roughly 20%, but this increase was significant only in the w/o/Ril group (*p* = 0.01). Again, in the w/o/Ril group only, the rate of weight loss was significantly worse. Secondly, the ALS parameters were compared at sNfL2 between the two ALS groups and none reached significance. sNfL2 levels between the two groups were comparable. The sNfL1 to sNfL2 delay was shorter in patients w/Ril: 8.3 ± 5.1 vs. 14.1 ± 12.3 months, but this was not significant.

**TABLE 2:** Characteristics of the two ALS subgroups, according to riluzole start

	w/riluzole at sNfL1	w/o riluzole at sNfL1	*p*‐value
*n*	86	55	
Gender, male/female (ratio)	52/34 (1.53)	36/19 (1.89)	ns
Bulbar onset, *n*	22	16	
Upper limb onset, *n*	29	17	ns
Lower limb onset, *n*	35	22	
Age of onset, years	61.3 ± 11.6	62.0 ± 11.4	ns
Usual weight, kg	71.4 ± 13.2	75.7 ± 14.5	ns
Characteristics at sNfL1			
Age, years	64.8 ± 11.5	64.1 ± 11.2	ns
Delay since onset, months	35.5 ± 35.6	18.7 ± 43.6	<0.0001
sNfL1, pg/ml	64.1 ± 45.8	64.3 ± 50.1	ns
ALSFRS‐R	36.8 ± 7.1	41.7 ± 5.2	<0.0001
ALSFRS‐R rate of decline	0.5 ± 0.4	0.7 ± 0.8	ns
Rate of % weight loss	0.19 ± 0.6	0.43 ± 0.9	ns
Characteristics at sNfL2			
Delay since sNfL1, months	8.3 ± 5.1	14.1 ± 12.3	ns
sNfL2, pg/ml	76.8 ± 71.2	78.8 ± 58.6	ns
*p*‐value, vs sNfL1	ns	0.001	
ALSFRS‐R	31.3 ± 8.7	33.8 ± 8.5	ns
*p*‐value, vs sNfL1 step	<0.0001	<0.0001	
ALSFRS‐R rate of decline	0.6 ± 0.7	0.7 ± 0.6	ns
*p*‐value, vs sNfL1 step	ns	ns	
Rate of % weight loss	0.05 ± 1.1	−0.12 ± 0.9	ns
*p*‐value, vs sNfL1 step	ns	0.003	

Abbreviations: ALSFRS‐R, amyotrophic lateral sclerosis rating scale‐revised. All rates are monthly rates; *n*, number of subjects; except for gender and site of onset, values are means ± SD; ns, not significant; sNfL, serum neurofilament light chain; w/ riluzole: patient group taking riluzole before sNfL1; w/o riluzole: patient group starting riluzole at sNfL1.

According to site of onset, sNf1 concentrations were higher in bulbar patients who were also older at entry (*p* = 0.012, Table S2). In all three subgroups (bulbar, upper limb and lower limb) sNfL2 was higher than sNfL1, but this was not significant for patients with upper limb onset of ALS. The largest and most significant increase in sNfL levels was recorded in bulbar patients. The bulbar group had the highest sNfL2 concentrations (*p* = 0.003). In this group, sNfL2 levels increased by 33% compared to 9% and 20% for patients with upper limb and lower limb, respectively.

### 3.3 Gender and rate of ALSFRS‐R decline are independently associated with sNfL levels

In an attempt to refine the analysis of the factors influencing sNfL variations with time, ALS clinical parameters were introduced in a linear regression model with uni‐ and multivariate analysis. At the time of sNfL1 measurement, while most factors were significantly associated with higher sNfL levels, only gender and ALSFRS‐R rate of decline were independently associated with higher sNfL1 levels (Table 3). While gender and ALSFRS‐R rate of decline were still the most significant parameters independently associated with higher sNfL2 levels, age at ALS onset and age at blood collection also showed statistical significance.

**TABLE 3:** Factors influencing sNfL concentrations in ALS patients

Variable	Univariate		Multivariate	
	HR (CI 95%)	*p*‐value	HR (CI 95%)	*p*‐value
sNfL1				
Gender	0.299 [0.139; 0.459]	0.0003	0.271 [0.115; 0.427]	**0.001**
Site of onset	−0.304 [−0.503; −0.105]	0.003	−0.046 [−0.233; 0.142]	0.629
Age at ALS onset	0.238 [0.073; 0.390]	0.005	2.781 [−3.030; 8.591]	0.346
Age at blood collection	0.142 [−0.025; 0.301]	0.093	−2.632[−8.382; 3.120]	0.367
Delay since onset	−0.267 [−0.428; −0.105]	0.001	0.565 [−1.072; 2.202]	0.496
ALSFRS‐R	−0.100 [−0.261; 0.067]	0.240	–	
ALSFRS‐R rate of decline	0.459 [0.310; 0.586]	1.5·10^−8^	0.221 [0.061; 0.382]	**0.007**
Rate of weight loss	0.103 [−0.064; 0.270]	0.224	–	
sNfL2				
Gender	0.343 [0.186; 0.501]	0.0003	0.316 [0.168; 0.464]	**0.00004**
Site of onset	−0.336 [−0.533; −0.139]	0.001	−0.127 [−0.306; 0.053]	0.165
Age at ALS onset	0.275 [0.112; 0.424]	0.001	0.874 [0.349; 1.399]	**0.001**
Age at blood collection	0.162 [−0.004; 0.320]	0.055	−0.760 [−1.287; −0.234]	**0.005**
Delay since sNfL1	−0.096 [−0.262; 0.071]	0.260	–	
ALSFRS‐R	−0.123 [−0.283; 0.044]	0.145	–	
ALSFRS‐R rate of decline	0.346 [0.187; 0.487]	0.00003	0.264 [0.118; 0.411]	**0.001**
Rate of weight loss	0.163 [−0.003; 0.328]	0.054	0.065 [−0.078; 0.208]	0.371

Abbreviations: ALSFRS, amyotrophic lateral sclerosis; ALSFRS‐R, amyotrophic lateral sclerosis rating scale‐revised; HR, hazard ratio; sNfL, serum neurofilament light chain.

### 3.4 Gender is associated with sNfL changes with time in ALS patients

Univariate and multivariate linear regression models were also used to determine the type of ALS parameters significantly and independently influencing sNfL variations between the two blood collections (Table 4). In univariate analysis, only gender and ALSFRS‐R rate of decline were significantly below the limit of 0.10. However, in multivariate analysis, only gender was independently associated with sNfL changes with time.

**TABLE 4:** Factors influencing sNfL change between sNfL1 and sNfL2 in ALS patients

Variable	Univariate		Multivariate	
	HR (CI 95%)	*p*‐value	HR (CI 95%)	*p*‐value
Gender	−0.258 [−0.420; −0.096]	0.002	−0.244 [−0.406; −0.82]	**0.003**
Site of onset	0.138 [−0.066; 0.342]	0.183	–	–
Age of ALS onset	−0.135 [−0.301; 0.031]	0.111	–	–
*sNfL1*				
Age at blood collection	−0.106 [−0.273; 0.060]	0.210	–	–
Delay since ALS onset	0.103 [−0.064; 0.270]	0.225	–	–
ALSFRS‐r	−0.058 [−0.226; 0.109]	0.492	–	–
ALSFRS‐R rate of decline	−0.158 [−0.324; 0.007]	0.061	−0.131 [−0.293; 0.031]	0.112
Rate of weight loss	−0.123 [−0.290; 0.043]	0.145	–	–
*sNfL2*				
Age at blood collection	−0.098 [−0.265; 0.069]	0.249	–	–
Delay since NfL1	0.132 [−0.034; 0.298]	0.118	–	–
ALSFRS‐r	−0.069 [−0.236; 0.098]	0.416	–	–
ALSFRS‐R rate of decline	−0.095 [−0.262; 0.072]	0.261	–	–
Rate of weight loss	0.077 [−0.090; 0.245]	0.362	–	–

Abbreviations: ALSFRS: amyotrophic lateral sclerosis; sNfL: serum neurofilament light chain; HR: hazard ratio; ALSFRS‐R: amyotrophic lateral sclerosis rating scale‐revised.

## 4 DISCUSSION

This study explored the evolution of sNfL levels at two different time points in 33 controls and in 141 ALS patients. As already known, sNfL concentrations were higher in ALS patients.^3^, ^4^, ^5^ With time, NfL values were unchanged in controls while they significantly increased in ALS patients, and in the w/o/Ril subgroup. Main determinants of this increase were gender and rate of decline of ALSFRS‐R score as shown by multivariate analysis.

NfL is known to be a reliable biomarker for ALS diagnosis, and the present results support previous works.^3^, ^10^, ^12^ As sNfL is prognostic of ALSFRS‐R decline, the increase in sNfL levels with time in ALS patients from the present cohort is not surprising but had not been quantified to date. Here, over a mean 10.6‐month interval, sNfL levels increased by 26%, while ALSFRS‐R score worsened by 16%, well paralleling sNfL changes. However, this is a global consideration and individual variations may exist.^9^, ^10^, ^11^ sNfL changes between the two measures are not simply time‐related changes because in controls, who match for age and gender with ALS patients, sNfL levels remained steady. This variation is thus considered as ALS‐related even though it cannot be excluded that with larger time intervals it could be somewhat different.

The increase in sNfL with time in ALS patients is significant in the w/o/Ril group only, while this group is the smaller in size (*n* = 55) vs. the w/Ril group (*n* = 88). Moreover, gender ratio, although non‐significant, is higher in the w/o/Ril group. While women have the highest sNfL levels, the opposite would have been expected. This difference between the ALS subgroups may suggest that Riluzole has a delayed effect or its effect may increase with time: the longer you are treated with the drug the more you are likely to have a slower disease process. We cannot draw any definite conclusion regarding this aspect, and further studies are warranted to refine such variations.

sNfL is now admitted as a reliable marker of ALS prognosis. Different conditions, related to a treatment effect, induce a lowering of sNfL levels in motor neuron diseases. This is the case for the SMN inducer Nusinersen in SMA.^8^ In ALS, a lowering of sNfL has been induced by the antisense oligonucleotide Tofersen, but also by a high‐caloric nutrition.^7^, ^19^ It was thus important to try to monitor sNfL concentrations in ALS patients after Riluzole treatment. However, not only sNfL1 levels were the same in the two groups w/ and w/o Ril, but the increase in concentrations at sNfL2 was also of the same magnitude in the two groups. Several reasons and biases may explain the present results. First, the measure of NfL in serum, even though well correlated with CSF levels, may not be sensitive enough to properly monitor the effect of Riluzole. Secondly, comparing the effect on sNfL concentrations of high‐caloric nutrition or Tofersen with the effect of Riluzole may not be appropriate. Indeed, the amplitude of effect between these three therapeutic approaches is not the same; it is also likely that the delays of action are different and thus the timing of changes in sNfL levels, if any, may be different. Thirdly, even though the groups w/ and w/o Ril were clinically comparable, their size was rather limited, and mean delay for sNfL2 is less than 1 year, thus may be a too short one. Conversely, it cannot be excluded that a more frequent sNfL monitoring, for example, quarterly or even monthly, could have been more accurate to show sNfL level changes. Interestingly, sNfL levels after Tofersen treatment in ALS start to decrease by day 57, with no further decrease and even an increase between day 106 and day 169.^7^ Fourth, we cannot exclude that the effect of Riluzole on survival may not be linked to an effect on neurodegeneration.

Gender is the main factor, in the multivariate analysis, that significantly influences sNfL changes with time in the present study. Influence of gender seems important to consider further, as women had significantly higher sNfL levels than men. This has already been described.^3^, ^9^ The reason for this may lie in the difference of gender ratio between ALS patients according to sites of onset. Most patients with bulbar onset of ALS are women, and bulbar onset is also associated with the worst prognosis of ALS.^1^ It would thus be interesting to study sNfL variations both according to site of onset and according to gender. However, one should note that both for NfL1 and NfL2 levels the women group in the present study is heterogeneous with high standard deviations. Subsequently, a much larger population would be needed to properly address the question of sNfL changes in all ALS subgroups to better analyze the particular influence of site of onset and gender.

In conclusion, the present study showed that sNfL levels increased with time in ALS patients but failed to capture Riluzole effect. There are important unmet needs for surrogate markers to monitor treatment response in ALS. To date, NfL is a good candidate to achieve this goal and the present results should be interpreted with caution. Further studies are warranted, using larger population samples, more frequent sampling and on a longer period of time to better analyze the interest of this sNfL in monitoring treatment effect in ALS.

## AUTHOR CONTRIBUTIONS

**Florence Esselin** and **Elisa De La Cruz** involved in investigation (equal), supervision (supporting), validation (supporting), and writing—review and editing (equal). **Christophe Hirtz** involved in investigation, resources (equal), validation (equal), and writing—review and editing (equal). **Laurent Tiers, Sébastien Alphandéry, and Léandra Baudesson** involved in data curation (equal), investigation (supporting), resources (equal), and writing—review and editing (equal).: **Guillaume Taieb** involved in writing—review and editing (equal). **William Camu** involved in conceptualization (lead), formal analysis (lead), investigation (equal), methodology (equal), project administration (equal), supervision (equal), validation (equal), visualization (lead), writing—original draft (lead), and writing—review and editing (equal). **Sylvain Lehmann** involved in conceptualization (equal), data curation (lead), investigation (lead), methodology (equal), project administration (equal), resources (equal), supervision (equal), validation (equal), visualization (supporting), writing—original draft (equal), and writing—review and editing (equal).

## CONFLICT OF INTEREST

None declared.

## Supporting information


Table S1

Table S2

## Data Availability

The data that support the findings of this study are available from the corresponding author upon reasonable request

## References

[1] Masrori P , Van Damme P . Amyotrophic lateral sclerosis: a clinical review. Eur J Neurol. 2020;27:1918‐1929. 32526057 10.1111/ene.14393 PMC7540334

[2] Verde F , Silani V , Otto M . Neurochemical biomarkers in amyotrophic lateral sclerosis. Curr Opin Neurol. 2019;32:747‐757. 31403480 10.1097/WCO.0000000000000744

[3] Thouvenot E , Demattei C , Lehmann S , et al. Serum neurofilament light chain at time of diagnosis is an independent prognostic factor of survival in amyotrophic lateral sclerosis. Eur J Neurol. 2020;27:251‐257. 31437330 10.1111/ene.14063

[4] Steinacker P , Feneberg E , Weishaupt J , et al. Neurofilaments in the diagnosis of motoneuron diseases: a prospective study on 455 patients. J Neurol Neurosurg Psychiatry. 2016;87:12‐20. 26296871 10.1136/jnnp-2015-311387

[5] Zucchi E , Bonetto V , Sorarù G , et al. Neurofilaments in motor neuron disorders: towards promising diagnostic and prognostic biomarkers. Mol Neurodegener. 2020;15:58. 33059698 10.1186/s13024-020-00406-3 PMC7559190

[6] Vacchiano V , Mastrangelo A , Zenesini C , et al. Plasma and CSF neurofilament light chain in amyotrophic lateral sclerosis: a cross‐sectional and longitudinal study. Front Aging Neurosci. 2021;13:753242. 34744694 10.3389/fnagi.2021.753242 PMC8569186

[7] Miller T , Cudkowicz M , Shaw PJ , et al. Phase 1‐2 trial of antisense oligonucleotide Tofersen for SOD1 ALS. N Engl J Med. 2020;383:109‐119. 32640130 10.1056/NEJMoa2003715

[8] Olsson B , Alberg L , Cullen NC , et al. NFL is a marker of treatment response in children with SMA treated with nusinersen. J Neurol. 2019;266:2129‐2136. 31123861 10.1007/s00415-019-09389-8 PMC6687695

[9] Lu CH , Macdonald‐Wallis C , Gray E , et al. Neurofilament light chain: a prognostic biomarker in amyotrophic lateral sclerosis. Neurology. 2015;84:2247‐2257. 25934855 10.1212/WNL.0000000000001642 PMC4456658

[10] Verde F , Steinacker P , Weishaupt JH , et al. Neurofilament light chain in serum for the diagnosis of amyotrophic lateral sclerosis. J Neurol Neurosurg Psychiatry. 2019;90:157‐164. 30309882 10.1136/jnnp-2018-318704

[11] Benatar M , Wuu J , Andersen PM , Lombardi V , Malaspina A . Neurofilament light: a candidate biomarker of presymptomatic amyotrophic lateral sclerosis and phenoconversion. Ann Neurol. 2018;84:130‐139. 30014505 10.1002/ana.25276 PMC11348288

[12] Benatar M , Zhang L , Wang L , et al. Validation of serum neurofilaments as prognostic and potential pharmacodynamic biomarkers for ALS. Neurology. 2020;95:e59‐e69. 32385188 10.1212/WNL.0000000000009559 PMC7371380

[13] Bensimon G , Lacomblez L , Meininger V . A controlled trial of Riluzole in amyotrophic lateral sclerosis. ALS/Riluzole study group. N Engl J Med. 1994;330:585‐591. 8302340 10.1056/NEJM199403033300901

[14] Lacomblez L , Bensimon G , Leigh PN , Guillet P , Meininger V . Dose‐ranging study of Riluzole in amyotrophic lateral sclerosis. Amyotrophic lateral sclerosis/Riluzole study group II. Lancet. 1996;347:1425‐1431. 8676624 10.1016/s0140-6736(96)91680-3

[15] Traynor BJ , Codd MB , Corr B , Forde C , Frost E , Hardiman OM . Clinical features of amyotrophic lateral sclerosis according to the El Escorial and Airlie house diagnostic criteria: a population‐based study. Arch Neurol. 2000;57:1171‐1176. 10927797 10.1001/archneur.57.8.1171

[16] Kimura F , Fujimura C , Ishida S , et al. Progression rate of ALSFRS‐R at time of diagnosis predicts survival time in ALS. Neurology. 2006;66:265‐267. 16434671 10.1212/01.wnl.0000194316.91908.8a

[17] Teunissen CE , Tumani H , Bennett JL , Berven FS , Brundin L , Comabella M , Franciotta D , Federiksen JL , Fleming JO , Furlan R , Hintzen RQ , Hughes SG , Jimenez CR , Johnson MH , Killestein J , Krasulova E , Kuhle J , Magnone MC , Petzold A , Rajda C , Rejdak K , Schmidt HK , van Pesch V , Waubant E , Wolf C , Deisenhammer F , Giovannoni G , Hemmer B Consensus guidelines for CSF and blood biobanking for CNS biomarker studies. Mult Scler Int 2011;2011:246412, 1, 9. 22096631 10.1155/2011/246412 PMC3195993

[18] Rissin DM , Kan CW , Campbell TG , et al. Single‐molecule enzyme‐linked immunosorbent assay detects serum proteins at subfemtomolar concentrations. Nat Biotechnol. 2010;28:595‐599. 20495550 10.1038/nbt.1641 PMC2919230

[19] Dorst J , Schuster J , Dreyhaupt J , et al. Effect of high‐caloric nutrition on serum neurofilament light chain levels in amyotrophic lateral sclerosis. J Neurol Neurosurg Psychiatry. 2020;91:1007‐1009. 32788256 10.1136/jnnp-2020-323372

