# Supplementary material for: Repeated neurofilament light chain measurements did not capture Riluzole therapeutic effect in amyotrophic lateral sclerosis patients
Source: CNS Neurosci Ther. 2022 Jun 25;28(10):1532–8. doi: 10.1111/cns.13894 (PMC9437233; doi:10.1111/cns.13894)
Supplement: Supplementary file 1 — Table S1 [file CNS-28-1532-s002.docx]

**Supp Table 1.** Diagnosis and characteristics of the non-ALS group

| **Diagnosis** | **n** | **M/F** | **Age** | **sNfL1 (pg/mL)** | **sNfL2 (pg/mL)** |
| --- | --- | --- | --- | --- | --- |
| Primary lateral sclerosis | 11 | 6/5 | 67.5 ± 6.1 | 35.7 ± 22.4 | 31.5 ± 21.8 |
| Progressive muscular atrophy | 10 | 7/3 | 70.2 ± 10.7 | 23.1 ± 16.8 | 21.8 ± 18.0 |
| C9ORF72 pre symptomatic | 1 | 0/1 | 45.6 | 23.0 | 12.1 |
| CBGD | 1 | 0/1 | 79.9 | 22.1 | 24.8 |
| Conversive motor disorder | 1 | 1/0 | 45.8 | 4.7 | 3.9 |
| Fronto-temporal dementia | 1 | 0/1 | 69.7 | 20.4 | 9.2 |
| Hereditary spastic paraplegia | 1 | 0/1 | 62.3 | 13.5 | 10.5 |
| Inclusion body myositis | 1 | 0/1 | 72.0 | 11.5 | 12.5 |
| Isaac’s syndrome | 1 | 1/0 | 63.7 | 17.6 | 16.3 |
| Kennedy’s disease | 1 | 1/0 | 60.9 | 5.0 | 5.7 |
| NMMCB | 1 | 1/0 | 55.5 | 15.5 | 14.0 |
| Post-polio syndrome | 1 | 0/1 | 73.3 | 21.0 | 31.3 |
| SCA3 cerebellar ataxia | 1 | 0/1 | 52.4 | 18.1 | 19.8 |
| Traumatic plexopathy | 1 | 0/1 | 50.3 | 9.0 | 7.8 |

For age and sNfL, values are mean ± SD, except when only one patient for the corresponding diagnosis. ALS, amyotrophic lateral sclerosis; M/F, male/female; sNfL, serum neurofilament light chain; CBGD, cortical basal ganglionic degeneration; NMMCB, motor neuropathy with multiple conduction blocks.
