# Supplementary material for: Repeated neurofilament light chain measurements did not capture Riluzole therapeutic effect in amyotrophic lateral sclerosis patients
Source: CNS Neurosci Ther. 2022 Jun 25;28(10):1532–8. doi: 10.1111/cns.13894 (PMC9437233; doi:10.1111/cns.13894)
Supplement: Supplementary file 2 — Table S2 [file CNS-28-1532-s001.docx]

**Supp Table 2**. ALS population characteristics according to site of onset

|  | **Bulbar** | **Upper limb** | **Lower limb** | *p* value |
| --- | --- | --- | --- | --- |
| **n** | 38 | 46 | 57 |  |
| **Gender, male/female** (ratio) | 17/21 (0.77) | 36/10 (3.60) | 35/22 (1.59) | ***0.006*** |
| **Age at onset, yrs** | 65.4 ±9.3 | 59.0 ±11.8 | 61.2 ±12.0 | ***0.036*** |
| **Usual weight, kg** | 70.6 ±13.5 | 75.5 ±12.5 | 72.9 ±14.9 | ns |
| ***Characteristics at NfL1*** |  |  |  |  |
| **sNfL, pg/mL** | 81.6 ±55.6 | 64.4 ±50.2 | 52.4 ±34.4 | ***0.012*** |
| **ALSFRS-R** | 39.1 ±6.7 | 39.5 ±7.5 | 37.8 ±6.3 | ns |
| **Weight, kg** | 67.2 ±13.2 | 73.6 ±12.5 | 70.9 ±14.0 | ns |
| *p* value, **vs usual weight** | ***<0.001*** | ns | ns |  |
| ***Characteristics at NfL2*** |  |  |  |  |
| **sNfL, pg/mL** | 108.2 ±72.4 | 70.4 ±73.2 | 63.0 ±48.4 | ***0.003*** |
| *p* value, **vs NfL1 data** | ***<0.001*** | ns | ***0.004*** |  |
| **ALSFRS-R** | 31.4 ±9.4 | 32.2 ±9.8 | 32.9 ±7.2 | ns |
| *p* value, **vs NfL1 data** | ***<0.0001*** | ***<0.0001*** | ***<0.0001*** |  |
| **Weight, kg** | 65.0 ±14.0 | 74.2 ±12.4 | 71.3 ±15.4 | ***0.012*** |
| *p* value, **vs NfL1 data** | ***0.005*** | ns | ns |  |

Values are means ± SD, sNfL, serum neurofilament light chain. ALSFRS-R: amyotrophic lateral sclerosis rating scale-revised
